# Spontaneous closure of a macular hole in an unusually long time after primary vitrectomy

**DOI:** 10.5935/0004-2749.20220055

**Published:** 2025-08-22

**Authors:** Julio Daniel Grigera, Tomás Castro Feijóo, Alberto D. Zambrano

**Affiliations:** 1 Fundación de Cirugía Ocular Jorge Zambrano, Autonomous City of Buenos Aires, Argentina

**Keywords:** Retinal perforations/surgery, Vitreoretinal surgery, Treatment failure, Time factors, Remission, spontaneous, Humans, Case reports, Perfurações retinianas/cirurgia, Cirurgia vitreorretiniana, Falha de tratamento, Fatores de tempo, Remissão espontânea, Humanos, Relato de caso

## Abstract

We report the case of a 68-year-old man who presented to our outpatient clinic for routine examination. Fifteen months before, he had undergone combined cataract and idiopathic full-thickness macular hole surgery in his right eye at another institution. In the present evaluation, the best-corrected visual acuity in his right eye was counting fingers. Fundus examination evidenced an idiopathic full-thickness macular hole in that eye, which was confirmed on spectral domain optical coherence tomography. A new surgery was offered, but the patient declined. Twenty-one months after his first consultation with us (36 months after the surgery), spectral domain optical coherence tomography revealed spontaneous closure of the idiopathic fullthickness macular hole, with a gap at the foveal ellipsoid zone. At the final visit, 22 months after the closure of the idiopathic full-thickness macular hole, the patient’s best-corrected visual acuity was 20/25, and the gap at the ellipsoid zone had decreased.

## Abstract (pt)

Este é o relato do caso de um homem de 68 anos que procurou nosso ambulatório para exames de rotina. Quinze meses antes, ele havia se submetido a uma cirurgia conjunta de catarata e buraco macular idiopático de espessura total em seu olho direito, em outra instituição. Durante a consulta em nosso ambulatório, a melhor acuidade visual corrigida no olho direito era de contagem de dedos. O exame do fundo evidenciou um buraco macular idiopático de espessura total naquele olho, o que foi confirmado por uma tomografia de coerência óptica de domínio espectral. Uma nova cirurgia foi oferecida, mas o paciente recusou. Vinte e um meses após sua primeira consulta (36 meses após a cirurgia), a tomografia de coerência óptica de domínio espectral revelou o fechamento espontâneo do buraco macular idiopático de espessura total, com uma lacuna na zona elipsoide foveal. Na última consulta, 22 meses após o fechamento do buraco macular idiopático de espessura total, a melhor acuidade visual corrigida foi de 20/25 e a lacuna na zona elipsoide havia diminuído.

## INTRODUCTION

An idiopathic full-thickness macular hole (iFTMH) is an opening of the whole retina at the fovea^(1)^. Vitrectomy with internal limiting membrane (ILM) peeling may achieve a nearly 100%-hole closure rate by the end of the first postoperative week^(2)^. We report a case of spontaneous closure of an iFTMH 36 months after the primary vitrectomy and its 22-month follow-up results.

## CASE REPORT

**Figure 1:**
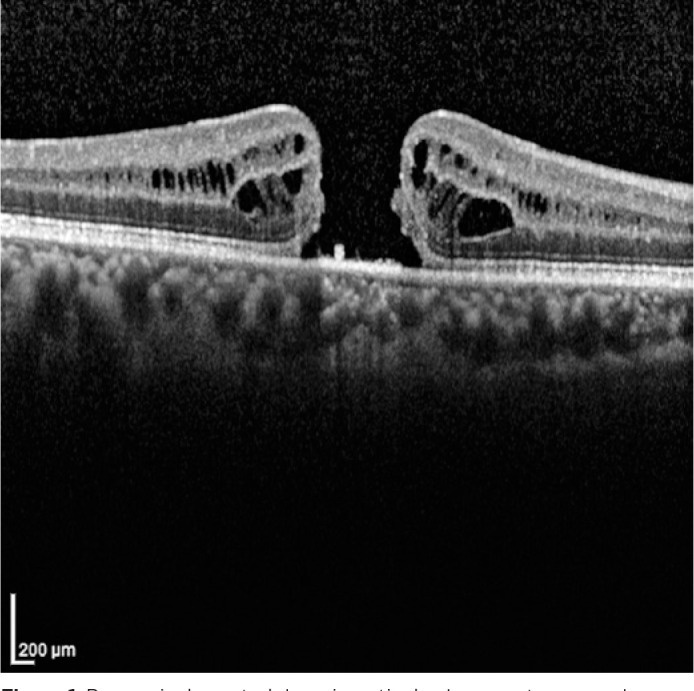
Presurgical spectral domain optical coherence tomography scan showing the idiopathic full-thickness macular hole.

**Figure 2:**
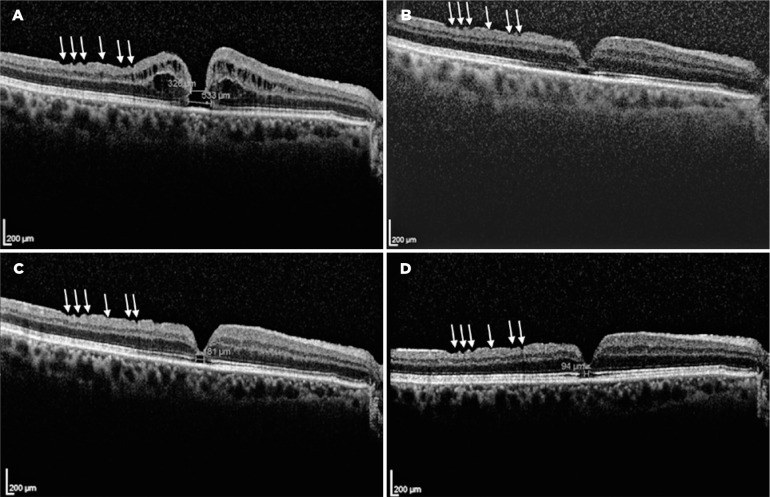
Postsurgical spectral domain optical coherence tomography scans. The idiopathic full-thickness macular hole (iFTMH) persisted for 15 months after primary vitrectomy (A). Its midand base-hole diameters were of 326 and 533 microns, respectively. The best-corrected visual acuity (BCVA) was counting fingers. iFTMH closure was verified 36 months after surgery (B). Posterior capsular opacification precluded obtaining a clear image, and the BCVA was 20/200. This improved when posterior capsulotomy was performed 12 months after (C). A 181-micron gap at the foveal ellipsoid zone was present, and the BCVA was 20/30. At the last visit, 22 months after the iFTMH closure (D), a 94-micron gap at the EZ remained. The corresponding BCVA was 20/25. The inner retinal dimples (white arrows) can be observed on the temporal side of the macular retinal nerve fiber layer.

A 68-year-old man who had undergone combined cataract and iFTMH surgery in his right eye (OD) 15 months before at another institution presented at our outpatient clinic for routine examination. The best-corrected visual acuity (BCVA) in OD was counting fingers, with pseudophakia and normal intraocular pressure. Fundus examination evidenced an iFTMH in the right eye; the left eye showed no abnormal findings. The patient’s presurgical spectral domain optical coherence tomography (SD-OCT) study demonstrated a FTMH, but the iFTMH was not measured (Figure 1). A new SD- OCT scan revealed the persistence of the iFTMH, with 326 and 533 microns of midand base-hole diameters, respectively (Figure 2A). A new surgery was offered, but the patient declined. Thus, he was set for follow-up every 6 months.

Twenty-one months after his first consultation with us (36 months after the surgery), the BCVA in the right eye had improved to 20/200. Slit-lamp examination revealed posterior capsule opacification while fundus examination and SD-OCT revealed the spontaneous closure of the iFTMH (Figure 2B), with a gap at the foveal ellipsoid zone (EZ).

Twelve months after the verified closure of the iFTMH, posterior capsulotomy was performed, and the BCVA improved to 20/30. At the final visit, 22 months after the closure of the iFTMH, the patient’s BCVA was 20/25, and the gap at the EZ diminished its diameter from 181 microns (Figure 2C) to 94 microns (Figure 2D).

## DISCUSSION

We report a case of spontaneous closure of an iFTMH 36 months after an unsuccessful primary vitrectomy. To the best of our knowledge, this is the longest time elapsed between primary vitrectomy and closure of the iFTMH reported in the literature.

Odrobina et al.^(3)^, Afrashi^(4)^, and Patel et al.^(5)^ published case reports of spontaneous delayed closure of new iFTMHs 1, 5, and 28 months after vitrectomy for vitreomacular traction, respectively. The ILM was peeled in the first case; however, in the second case, it was dissected but not removed because of alleged tight adhesions at the macula. By contrast, in the latter, ILM peeling was not even attempted.

Two case reports by Falkner-Radler et al.^(6)^ and Lee et al.^(7)^ demonstrated the late spontaneous closure of a pre-existing iFTMH 9 months after unsuccessful vitrectomy, both with ILM peeling and C_3_F_8_ gas tamponade. The closure of the latter was associated with the development of a type 1 neovascular membrane that apparently brought together the retinal tissue.

Rishi et al.^(8,9)^ reported 2 cases of spontaneous closure of traumatic macular holes 7 months after vitrectomy with ILM peeling and gas tamponade. One of the cases^(9)^ was associated with submacular hemorrhage. The authors presumed that physical approximation of the hole edges could have been facilitated by submacular blood removal. They affirmed that a dynamic process occurring at the macula long after the surgery may be responsible for the change in configuration of the macular hole. None of the cited papers explained the long time elapsed before the hole closure.

A pre-existing iFTMH was the primary reason for the surgery in our patient. We did not have access to the preoperative clinical records, but ILM peeling was assumed to be performed owing to the presence of superficial focal retinal depressions known as inner retinal dimples (Figure 2, white arrows), which are supposed to be due to diffuse loss of the Müller cell end-feet and whose presence is considered a late sign of an already peeled ILM^(10)^. The preoperative SD-OCT study result brought to us by the patient was from another clinic, and unfortunately, the iFTMH was not measured. Thus, the presurgical and postsurgical iFTMH sizes could not be compared.

For personal reasons, our patient chose not to undergo another surgery. Although prospective studies are lacking, the current retrospective evidence holds that 71% to 84% of iFTMHs close with a secondary procedure. This may be achieved with different techniques, but the available evidence does not show superiority of one procedure over the others^(11)^.

Sokol et al.^(12)^ presented a multicenter, retrospective observational case series of 14 patients who underwent successful off-label topical treatment with steroids, nonsteroidal anti-inflammatory drugs, and carbonic anhydrase inhibitors for closure of iFTMHs. They suggested that this treatment could trigger both decreased inflammation and increased fluid absorption through the retinal pigment epithelium, thereby closing the hole.

However, the possibility that treated iFTMHs could have closed spontaneously without treatment must be considered. This study has no arm for comparison or data on treated patients whose holes did not close. Hence, further investigation is needed.

When the iFTMH closed spontaneously and the BCVA improved to 20/200, capsular opacification from the original combined procedure precluded further visual improvement for 12 months, until capsulotomy was performed. The facts that EZ gradually regenerated up to a 94-micron gap and that BCVA improved to 20/25 at the last follow-up visit are consistent with the evidence of long-term postoperative vision improvement associated with the restoration of the morphological features of the outer retinal layers^(13)^.

Bringmann et al.^(14)^ previously hypothesized that the mechanism of spontaneous iFTMH closure is likely mediated by an annular contraction of the horizontal Müller cell side processes in the foveal outer plexiform layer (OPL) and the Müller cell structures that envelop the photoreceptor cells at the external limiting membrane. These movements result in a centripetal shift of the foveal walls and the closure of the hole at the level of the OPL/inner part of the outer nuclear layer (ONL). After the closure of the hole, the EZ regenerates, possibly due to a centripetal displacement of the photoreceptor cells around the EZ defect and/or an outgrowth of the newly formed photoreceptor segments from the photoreceptor cell somata in the central ONL.

In conclusion, we report the unusual occurrence of delayed spontaneous closure of an iFTMH. Through the 22-month follow-up after the iFTMH closure, anatomical and functional improvements were detected. When the patient rejects a secondary procedure, it could be acceptable to assess the macula with serial OCTs scans considering the possibility of spontaneous delayed closure and anatomical regeneration of the EZ.
